# Observations upon the Inutility of the Abdominal Bandage after Parturition

**Published:** 1852-06

**Authors:** F. C. T. Arnoldi

**Affiliations:** Lecturer upon Midwifery, St. Lawrence School of Medicine, Montreal, &c., &c.


### SELECTIONS

FROM

### FOREIGN AND HOME MEDICAL JOURNALS.

---

*Observations upon the Inutility of the Abdominal Bandage after Parturition, being part of a lecture delivered this session.* By F. C. T. Arnoldi, M. D., Lecturer upon Midwifery, St. Lawrence School of Medicine, Montreal, &c., &c.—Having now told you all the essential points to be rigidly attended to during the process of labor, you must be made as familiarly acquainted with the nursing part of the puerperal state. The child having been disconnected from the mother and the placenta withdrawn, your next care should be, that the uterus has assumed a state of permanent contraction, and for this purpose, you should diligently watch, for at least half an hour, because, very alarming symptoms may supervene, such as uterine hemorrhage, syncope, or convulsions. The most common is uterine hemorrhage. This may take place under various circumstances, but the most ordinary, is an atonic state of the uterus, the cause of this condition may either be immediate or remote, that is to say, immediately after delivery, the uterus may cease to contract, or having, to all appearances, permanently contracted, it may relax, get into the atonic condition, and so give rise to profuse hemorrhagic discharge. Now this discharge, in both instances, is owing to the *baillant* condition of the uterine venous sinuses; fortunately, however, this is not an every day occurrence, yet apparently, with a view of anticipating such a serious misfortue, our ancestors and modern authors lay down strict injunctions for the application of an abdominal roller or bandage. There was a time, when I would have thought it almost sacrilegious to have acted in contravention to this precept—but a case happened to come under my charge, in which I was very much interested, and which gave rise to close anatomical investigation on my part. I shall narrate it to you in a familiar manner, and show you the conclusions I came to:

Mrs. A. was confined on the 14th August, 1830. Being a prima para, it was, as usual, a painful and somewhat tedious case, but on the whole nothing uncommon, the secretion of milk set in within fifty-four hours, and nothing appeared to indicate the prohibition to her sitting up in an arm chair on the fourth day, for the purpose of having her bed made; the bandage had been applied round the abdomen according to orthodox rules. On the evening of the fourth day, she complained bitterly of pains in her loins, and continual bearing down pains—supposing that the bandage had not been applied sufficiently tight, it was drawn a little tighter, but instead of affording any relief, the pains were increased. It then struck me, that as the bowels were in good order, the bandage was the whole and sole cause of the evil, and, to satisfy myself, I examined a skeleton very attentively, and I came to the conclusion that my notion was correct; in proof, gentlemen, only look at this skeleton, and observe (that which I was so careful to impress on your minds in the former part of my course) the very obtuse angle, the brim of the pelvis bears to the axis of the spinal column. You see, that the promontory of the sacrum is several inches above the horizontal line of the pubis—that the promontory projects forwards—the sacrum recedes, and thereby forms a recipient cavity. Again, gentlemen, remark the lateral configuration of the skeleton, and you perceive that the great projecting alæ ilii, the crests of which are on a line almost parallel with the promontory of the sacrum, form the point from which the tapering figure starts upwards towards what is called the waist; this you see is perfectly demonstrable even on the skeleton, how much more so is it as you must have observed, on the soft subject? Well, having satisfied myself on these two points, I first of all came to the conclusion that no well-formed woman could keep an abdominal bandage in its proper place, unless, indeed, it were very tightly put on, and then I inferred that if very tightly put on, it must act very detrimentally on the yet heavy uterus. The bandage you see, to keep its place, so as to act upon the uterus, must be applied over the hip, otherwise, it must act upon the abdominal viscera, and make them press down upon the uterus, in which case the bandage necessarily slips up to the small diameter of the waist, and can no longer carry out the original object intended, and proves a sore annoyance to your patient, who, believing there is some charm in it, keeps herself in a constant fidget by pulling it down, and pulling it, as she believes, into the right place; now, this of itself, gives rise to much unnecessary muscular exertion. If the bandage be kept tight over the hips, it must necessarily act upon the uterus, and that, in two ways, presuming it in the first place to be necessary, it must be for the purpose of exciting the uterus to contraction, or it must be for the purpose of arresting uterine hemorrhage, but I maintain that it can produce neither the one nor the other effect; in the first place: it mechanically presses the yet heavy uterus into the bottom of the sacrum (which affords it every facility to descend,) and thereby lays the first seeds for prolapsus uteri, and in the second place, if uterine hemorrhage do supervene, it is one of the most fallacious resosts you can trust to; but further, I will tell you when I come upon that subject. To come back to my case, Mrs. A., as I told you before, suffered much from bearing down pains on the fourth day, and I may now add, she continued to do so for many months after; time rolled on and still the pains continued, until fortunately she again got in the family way, but even then, and for the four first months, she was constantly threatened with a miscarriage. She, however, had the good luck to go on to her full time. I delivered her the second time, and determined not to use the bandage again, in lieu of which, as a matter of precaution, I exacted a little more bed rest. My orders were strictly attended to, and her recovery was all that could be wished for. From that day to this, she has had no recurrence of bearing down pains after her confinements, though similarly treated, notwithstanding her having had ten more children, making twelve in all. Since my experience in this case, which was in 1832, (her second confinement,) I have never applied the abdominal bandage in my private or hospital practice, I have never met with any sinister results from its omission, and I know of other medical men who have often followed my example equally satisfactorily. Uterine hemorrhage is the great bugbear, and certainly it is a most serious concomitant, but when it does take place, I can assure you, gentlemen, your patient would be badly off, if you had nothing else to trust to but the abdominal bandage. Obesity, or the becoming (to use vulgar parlance) "pot-bellied," is the next argument against the omission of the bandage, but I can assure you, that in the whole of my practice, I can not trace one single instance to such a cause. Hear what a lady writes to me from Quebec: "Dear Doctor, I was confined on ———, and I determined on following your instructions to the letter. My physician and nurse thought I was mad, nevertheless, I maintained my point, and certainly, I have every reason to be grateful to you, for besides having made a much more favorable recovery than usual, I have been relieved of that horrible annoyance—*the belly band; and the bearing down pains have not returned.*"

Again, gentlemen, let us look at the puerperal state in a strictly pathological and physiological point of view. Is not parturition strictly and simply a natural and healthy process? Certainly it is, except under casual circumstances. Can the distension of the abdomen from utero-gestation be compared with the abdominal distension from ascites? No! In the one instance, you have a healthy tonic, in the other, you have one of the worst forms of unhealthy atonic action; in the one you have a fixed duration, at the end of which tonic muscular contraction sets in, and the abdominal parietes resume their normal condition; in the other, the letting out of the fluid by the trocar is followed by a mere collapse of the abdominal parietes, so that the capacity of the abdomen would still remain the same, unless a roller bandage were applied. Were I now speaking of uterine hemorrhage, I would point out to you, how thoroughly insufficient the abdominal bandage alone would be; at any rate, I would show you how much you would be mistaken, if, under such circumstances, you trusted to it, as your main stay. Talking of uterine hemorrhage, it is a very singular fact, that during a practice of 25 years, I have never met with more than one case, except such as I have seen in consultation, and of that case I was forewarned, as my patient had suffered from it on three former occasions. Notwithstanding, gentlemen, what I have told you as the result of my own practice, I must warn you against being too dogmatic in the course of yours Old women must have their way, and their ways are almost always based upon prejudice, you therefore should be prepared and willing to consent to their notions. If you perceive a very especial desire for the application of the abdominal bandage, my advice to you is, by all means, to consent to it; it is only necessary for you to see that it is not put on so tight as to endanger your patient to future uterine inconveniency.

*Canada Journal.*

